# Non-coding single nucleotide variants affecting estrogen receptor binding and activity

**DOI:** 10.1186/s13073-016-0382-0

**Published:** 2016-12-13

**Authors:** Amir Bahreini, Kevin Levine, Lucas Santana-Santos, Panayiotis V. Benos, Peilu Wang, Courtney Andersen, Steffi Oesterreich, Adrian V. Lee

**Affiliations:** 1 Deparmtent of Human Genetics, University of Pittsburgh, Pittsburgh, PA USA; 2 Department of Pharmacology and Chemical Biology, University of Pittsburgh Cancer Institute, Pittsburgh, PA USA; 3 Womens Cancer Research Center, Magee-Women Research Institute, Pittsburgh, PA USA; 4 Department of Pathology, University of Pittsburgh, Pittsburgh, PA USA; 5 Department of Computational and Systems Biology, University of Pittsburgh, Pittsburgh, PA USA; 6 Department of Biomedical Informatics, University of Pittsburgh, Pittsburgh, PA USA; 7 School of Medicine, Tsinghua University, Beijing, 100084 People’s Republic of China; 8 AstraZeneca, Oncology iMED, 35 Gatehouse Drive, Waltham, MA USA

**Keywords:** Breast cancer, Estrogen receptor, DNA binding, IGF1R, Non-coding SNVs

## Abstract

**Background:**

Estrogen receptor (ER) activity is critical for the development and progression of the majority of breast cancers. It is known that ER is differentially bound to DNA leading to transcriptomic and phenotypic changes in different breast cancer models. We investigated whether single nucleotide variants (SNVs) in ER binding sites (regSNVs) contribute to ER action through changes in the ER cistrome, thereby affecting disease progression. Here we developed a computational pipeline to identify SNVs in ER binding sites using chromatin immunoprecipitation sequencing (ChIP-seq) data from ER+ breast cancer models.

**Methods:**

ER ChIP-seq data were downloaded from the Gene Expression Omnibus (GEO). GATK pipeline was used to identify SNVs and the MACS algorithm was employed to call DNA-binding sites. Determination of the potential effect of a given SNV in a binding site was inferred using reimplementation of the is-rSNP algorithm. The Cancer Genome Atlas (TCGA) data were integrated to correlate the regSNVs and gene expression in breast tumors. ChIP and luciferase assays were used to assess the allele-specific binding.

**Results:**

Analysis of ER ChIP-seq data from MCF7 cells identified an intronic SNV in the IGF1R gene, rs62022087, predicted to increase ER binding. Functional studies confirmed that ER binds preferentially to rs62022087 versus the wild-type allele. By integrating 43 ER ChIP-seq datasets, multi-omics, and clinical data, we identified 17 regSNVs associated with altered expression of adjacent genes in ER+ disease. Of these, the top candidate was in the promoter of the GSTM1 gene and was associated with higher expression of GSTM1 in breast tumors. Survival analysis of patients with ER+ tumors revealed that higher expression of GSTM1, responsible for detoxifying carcinogens, was correlated with better outcome.

**Conclusions:**

In conclusion, we have developed a computational approach that is capable of identifying putative regSNVs in ER ChIP-binding sites. These non-coding variants could potentially regulate target genes and may contribute to clinical prognosis in breast cancer.

**Electronic supplementary material:**

The online version of this article (doi:10.1186/s13073-016-0382-0) contains supplementary material, which is available to authorized users.

## Background

Breast cancer is a major public health issue with an increasing incidence over the past decade in the US. Endocrine therapy, such as the antiestrogen tamoxifen and aromatase inhibitors, are the most successful treatment for breast cancer in which estrogen signaling is active. Estrogen signaling is mediated through estrogen receptors (ER), which upon binding the ligand estradiol, is recruited to DNA at estrogen response elements (EREs) and alters transcription of downstream target genes essential for cell growth and proliferation. The development of chromatin immunoprecipitation (ChIP) assays has allowed a genome-wide analysis of ER ChIP-binding sites. For example, ER binds different sites in tamoxifen responsive versus resistant cell lines and tumors [1]. However, the potential genomic changes underlying unique ER ChIP-binding sites in different models are still unclear.

A number of studies indicate that single nucleotide polymorphisms (SNPs), referred to as germline polymorphism, associated with breast cancer lie within EREs, such as those in FGFR2 and NRCAM [2–4]. In an in silico study, breast cancer risk-associated SNPs were enriched in ER ChIP-binding sites in a cell-type specific manner [5]. After analyzing these statistically significant SNPs in ER ChIP-binding sites, the authors found a variant suppressing the expression of a downstream gene, TXO3, via modulation of FOXA1 binding to DNA [5]. Clinical studies have also shown that regulatory SNPs in putative EREs can alter endocrine response to anti-estrogen drugs. A genome-wide association study (GWAS) of breast cancer patients in a phase III trial comparing anastrozole versus exemestane identified a SNP in the second intron of *ZNF423* that is associated with recruitment of ER in the presence of 4-hydroxytamoxifen [6]. A regulatory SNP was also identified which created an ERE conferring estrogen induction of TCL1A gene expression [7]. These data suggest a role for genomic variation underlying unique ER binding which may affect disease progression and response to anti-estrogen therapy.

ChIP followed by high-throughput sequencing is a powerful technique for genome-wide mapping of protein–DNA interactions [8]. Owing to the tremendous technological developments and reduction in the costs of the massively parallel sequencing (MPS), the number of ChIP-sequencing (ChIP-seq) studies has grown rapidly. ChIP-seq is generally utilized to characterize the binding sites of a specific protein through enrichment of the sequencing reads over the genome. Sequencing reads have generally been used to identify binding sites and the strength of binding; however, recent studies have examined the actual sequences themselves, to identify variants that affect DNA binding. BCRANK is an algorithm designed to detect regulatory SNPs (regSNPs) in ChIP-chip data based upon SNP genotyping in DNA-binding sites [9]. More recently, another strategy used ChIP-seq data to nominate regSNPs using the assumption that the enrichment of SNPs within transcription factor (TF) binding sites indicates their regulatory function [10]. This approach was applied to ENCODE data resulting in the characterization of a panel of SNPs associated with a number of transcription factors. Also, a new tool has been developed that identifies allele-specific binding of transcription factors from aligned ChIP-Seq reads at heterozygous SNVs [11]. These studies, however, lack a connection between regSNPs and the expression of *cis* target genes, which eventually determine the phenotypic output. Furthermore, appropriate motif detection could fine-tune the detection of biologically relevant variants in genome-wide binding sites.

Here we describe a strategy integrating computational and experimental approaches to detect and validate regulatory single nucleotide variants (regSNVs) defined as germ-line or somatic single base pair changes that can affect TF binding to DNA. Our framework interrogates ChIP-seq reads and nominates regSNVs affecting transcription factor binding motifs. Using the MCF7 cell line as the most studied model in breast cancer, we addressed whether ER binding is associated with regSNVs resulting in differential expression of downstream genes. We further applied our computational framework to all publicly available ER ChIP-seq data including ER-positive cell lines and tumors. Our strategy is able to identify genomic variation localized in TF binding sites having potential phenotypic significance.

## Methods

### Extracting genomic variants from ChIP-seq reads

ChIP-seq data were downloaded from the Gene Expression Omnibus (GEO), SRA, and ArrayExpress databases with the following accession numbers: GSE32222, GSE51022, GSE23701, GSE23893, SRA010193, E-TABM-828, GSE24166, GSE18046, GSE14664, and E-MTAB-223.

SNVs were identified from ChIP-seq data using the GATK pipeline (v2.4) [12]. Briefly, BWA (v0.7.5) was first employed to align the raw sequence reads to the human genome reference (hg18) using default options [13]. To increase the sequence read coverage over the binding regions for more accurate variant calling, reads from all the datasets on the same cell line were pooled (Additional file 1: Table S1). The reads were sorted and duplicates were removed using PICARD (v1.12) tools (ww.github.com/broadinstitute/picard). To refine the mapping quality, reads were locally realigned around the known indels and finally base calls were recalibrated using GATK tools by default options. The SNVs were identified by the GATK UnifiedGenotyper tool and known variants were annotated using dbSNP and 1000 Genome databases. Sequence calls with a coverage < 10 reads and/or a phred-score < Q20 and SNVs which were not within binding sites were filtered out by custom perl scripts.

### Identifying predicted DNA-binding sites using ChIP-seq data

The Model-based Analysis of ChIP-Seq (MACS) [14] was used to analyze all ER ChIP-seq data in breast cancer prior to July 2014 (Additional file 1: Table S1). MACS models the length of ChIP-seq reads to improve the resolution of predicted binding sites. A *p* value cutoff of 1e-5 was used and genome size which matches UCSC human hg18 assembly was used. In datasets which had sequenced untreated genomic DNA as a control, we used this sequence as input (untreated) control. MACS automatically calculates the tag size based on the reads length in the treatment file. Peak calling was performed in each ChIP-seq dataset first and binding sites from the same cell line were pooled.

### Motif analysis and *p* value scoring of the regSNVs

For each identified SNV, sequences containing reference allele and alternative allele were created in silico. Each sequence was independently scanned using the *ESR1* human position-specific matrices (PWM) based on JASPAR and TRANSFAC matrices database (JASPAR ID: MA0112.2 and TRANSFAC ID M02261) [15, 16]. Determination of the potential effect of a given SNV in a binding site was inferred using reimplementation of the is-rSNP algorithm [17]. Briefly, the is-rSNP calculates the background distribution of PWM scores, for a given PWM. Sequences containing reference and mutated alleles are scored and a *p* value for each score is calculated. The ratio of reference and mutated sequence *p* values are calculated and compared to the background distribution of *p* value ratios. If the *p* value obtained from the background distribution is less than 0.05, then a SNV is considered to affect a binding site. The SNVs are next ranked based on the adjusted *p* value ratio, which shows the significance of motif binding change after the introduction of the variant allele in the consensus sequence.

### Generating a list of estradiol (E2)-regulated genes

We sought to generate a master list of estrogen-regulated genes in breast cancer cells by querying publicly available array data. Studies were identified by searching the GEO. Search terms included “estradiol,” “estrogen,” “E2,” “breast cancer + E2,” and other variations to locate as many studies as possible. Initially, all studies found with vehicle (vhc) and estradiol (E2) treatment groups were compiled into a master list. We applied a data freeze to this list on 1 July 2013. Subsequently, we filtered out studies that only had one biological replicate due to lack of statistical power. We further narrowed the list by removing studies where estrogen treatment was > 24 h to focus on direct targets of ER. To confirm that these ER targets were also estrogen-regulated in vivo, we overlapped the union of estrogen-regulated genes from the in vitro studies with an MCF-7 xenograft study and with breast tumor data from The Cancer Genome Atlas (TCGA). For all in vitro and in vivo studies, estrogen-regulated genes were determined by downloading the raw data from the GEO and comparing estrogen and vhc treatments. Estrogen-regulated genes were considered those significantly different in estrogen treatment groups (*p* < 0.001). For TCGA data, estrogen-regulated genes were defined as those whose expression in ER+ versus ER– tumors was significantly different (*p* < 0.001). Significance was determined by unpaired, two-tailed t-tests. Our master list (Additional file 1: Table S2) comprises the intersection of estrogen-regulated genes in vitro, in vivo, and in TCGA data. The final list of studies we included can be found in Additional file 1: Table S3.

### TCGA and survival data analysis

SNP array data for 501 TCGA breast cancer cases was extracted from the Pittsburgh Genome Resource Repository (PGRR) (http://www.pgrr.pitt.edu/pgrr). These data were combined with TCGA gene expression profiles downloaded from the GEO (GSE62944) [18] for 1095 primary breast cancer samples. ER+ disease was defined by immunohistochemistry (IHC) staining annotated in TCGA data. The closest 3′ and 5′ genes to regSNVs were nominated as regSNV target genes. To analyze the correlation between regSNV and target gene expression, the log2 transcripts per million (TPM) expression was downloaded from preprocessed data [18] and compared between wild-type (WT) and variant carriers in ER+ tumors using the Mann–Whitney *U* test followed by a multiple comparison correction using Benjamini–Hochberg.

For the survival analysis of GSTM1, patients with ER+ tumors from the Molecular Taxonomy of Breast Cancer International Consortium (METABRIC) data were used under the IRB protocol (PRO16020311). Data from the KM-Plotter database were accessed via kmplot.com [19]. High expression of GSMT1 (METABRIC: Illumina probe 1762255, KM-Plotter: Affymetrix probe 204550_x_at) was defined by the upper quartile of GSMT1 expression among patients with ER+ tumors.

### ChIP

ChIP experiments were performed as previously described by our group [20]. Briefly, hormone-deprived cells were treated with 10nM E2 or vehicle (EtOH) for 45 min. We used ERα (HC-20) and rabbit IgG (sc2027) antibodies (Santa Cruz Biotechnologies) for immunoprecipitation. IgG was used as the negative control for immunoprecipitation. ChIP DNA was analyzed by qPCR using primers amplifying the rs62022087 locus in *IGF1R* (Additional file 1: Table S4).

### Allele-specific ChIP

ChIP DNA was first amplified by primers amplifying the region around the SNV site (Additional file 1: Table S4). Polymerase chain reaction (PCR) products were TA-cloned into pCR™4-TOPO® (Invitrogen) and plasmid was transformed to competent cells according to the manufacturer’s instructions. Thirty bacterial colonies were picked, DNA isolated, and subjected to Sanger sequencing. The WT and variant alleles were counted and the statistical significance of allele enrichment was determined by Chi-square test.

### RNA extraction and quantitative PCR (qPCR)

RNA was extracted using Illustra RNAspin Mini kit (GE Health). iScript master mix (Bio-Rad) for cDNA conversion and qPCR reactions were set up on a CFX384 thermocycler (Bio-Rad) at an annealing temperature of 60 °C for 40 cycles.

### Cloning and luciferase assay

ER ChIP binding sites with *IGF1R* SNP and WT alleles were amplified from MCF7 DNA using primers containing the restriction sites for EcoRV and HindIII (Additional file 1: Table S4). PCR products and backbone plasmid pGL4-TATA-luc (pGL4.23 from Promega) were digested and ligated using thermoscientific rapid DNA ligation kit and transformation using TOP10 competent cells. The plasmids were isolated using QIAprep Spin Miniprep Kit and further validated by Sanger sequencing.

MCF7 cells were grown in DMEM, supplemented with 10% fetal bovine serum (FBS). Before transfection, cells were estrogen deprived for 3 days with IMEM containing 10% charcoal-stripped FBS. Cells were transfected with pGL4– ER ChIP binding site (IGF1R) -TATA –luc containing WT or SNP allele and renilla using Lipofectamine LTX with Plus. A total of 10 nM E2 was added to media 24 h after transfection. Firefly and renilla luciferases were measured sequentially using the Dual-Luciferase Reporter Assay System (Promega).

## Results

### In silico identification of regSNVs in MCF7 ER ChIP-seq data

**Fig. 1:**
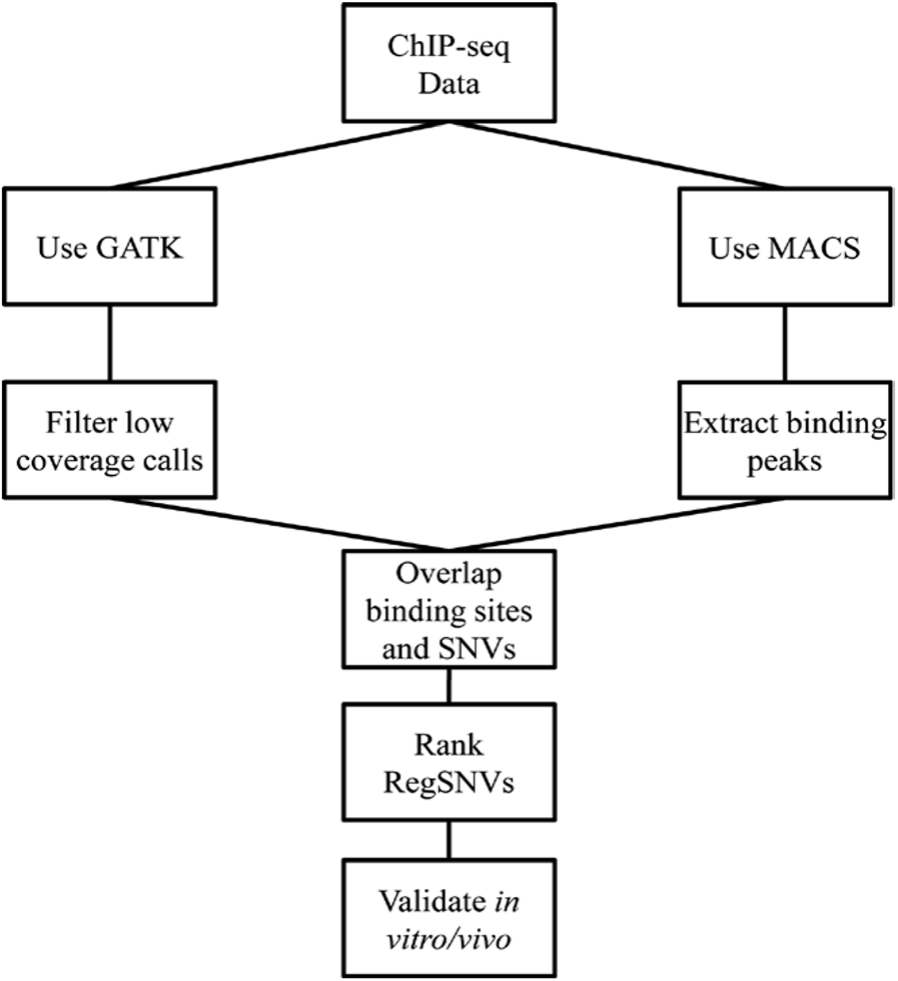
Analysis pipeline for detecting regulatory SNVs from ChIP-seq data. The above pipeline was utilized to extract and rank regSNVs based on their impact on the corresponding TF binding. MACS and GATK tools were recruited to identify binding sites and SNVs, respectively. The SNVs and binding peaks were overlapped and then regSNVs were ranked depending on how they alter EREs. One of the top candidates was selected for further functional studies

MCF7 is one of the most employed cell lines for studying molecular genetics of breast cancer [21]. Therefore, we selected publicly available ER ChIP-seq data from MCF7 (11 datasets) to identify regSNVs in ER ChIP-binding sites. Our computational approach (Fig. 1) consisted of: (1) identifying SNVs from MCF7 ER ChIP-seq data; (2) identifying ER ChIP-binding sites using MACS; (3) overlapping SNVs with ER ChIP-binding sites; and (4) rank regSNVs based upon the predicted alteration of motif binding.

We applied our computational workflow to nine ER ChIP-seq datasets from five different studies of MCF7 cells performed under similar experimental conditions (Additional file 1: Table S1) [1, 22–25]. The datasets were merged by combining the reads and 303,964,039 sequencing reads were mapped to the human genome (hg18) and identified a total of 1,409,406 SNVs and short indels. However, only 163,502 (11.6%) variants had sufficient coverage to pass filtering (see “Methods”) and were included in the final list for the analysis.

In parallel to SNV discovery, we used the MACS algorithm [14] to map genome-wide ER ChIP-binding sites using the same nine ER ChIP-seq datasets from above and analyzing each dataset independently. The results showed a wide range of variability in the number of binding peaks from 15,677 to 79,978 sites. To build a consensus peak list, we overlapped the ChIP binding sites of all datasets and selected the genomic regions which were common in at least six datasets. This led to the detection of 22,143 ER ChIP-binding sites with an average length of 385 bp. Using this panel of ER binding peaks, we next identified the SNVs which altered consensus EREs.

Motif assessment was performed by comparing ER-binding probabilities in the presence and absence of SNVs. The variants that were associated with a statistically significant change (see “Methods”) were selected as putative regSNVs. Our pipeline nominated 4019 motif-altering regSNVs (out of 163,502 variants), among which 2084 (52%) and 1935 (48%) variants were computationally predicted to increase and decrease the binding affinity of their corresponding motifs, respectively (Additional file 1: Table S5). To further refine the list, regSNVs were annotated with the closest adjacent genes and this list was compared to a list of estrogen-regulated genes derived from in vitro, in vivo, and TCGA data (see “Methods”). We focused on regSNVs capable of increasing ER binding and being within the proximity of an E2-regulated gene (<5 kb of distance) (Table 1). Interestingly, ten highly ranked statistically significant putative regSNVs (*p* < 1.0E-03) appeared close to genes previously shown to be oncogenic in breast cancer such as PVT1 [26], IGF1R [27], and GREB1 [28]. Of these, rs62022087, located in *IGF1R*, was identified by both JASPAR and TRANSFAC matrices, thus increasing the confidence of the call. Moreover, Sanger sequencing showed that this regSNV is heterozygous in MCF7, making it an appropriate candidate for allele-specific binding assays. This prompted us to investigate regulatory function of rs62022087 through further in vitro studies.

**Table 1:** Top regulatory SNVs promoting ER binding in proximity of E2-regulated genes in MCF7 cell line

Chr location (hg18)	Annotation	Gene	SNV ID	Database	Adjusted *p* value
chr8:128992864	ncRNA	PVT1	NA	TRANSFAC	2.26E-06
chr10:94821513	Intergenic	CYP26C1;CYP26A1	rs68040629	TRANSFAC	1.10E-05
chr15:97136484	Intronic	IGF1R	rs62022087	TRANSFAC, JASPAR	2.03E-05
chr10:121292409	Upstream	RGS10	rs10787978	TRANSFAC	3.39E-05
chr6:157157941	Intronic	ARID1B	rs12208040	TRANSFAC, JASPAR	3.63E-05
chr11:20014669	Intronic	NAV2	rs10741810	TRANSFAC, JASPAR	3.65E-05
chr17:54818764	Intronic	YPEL2	rs8073731	TRANSFAC, JASPAR	5.44E-05
chr2:10384622	Intronic	HPCAL1	rs2014889	TRANSFAC, JASPAR	5.62E-05
chr4:3456949	Intronic	DOK7	rs916189	TRANSFAC, JASPAR	1.09E-04
chr2:11712184	Intergenic	GREB1;NTSR2	rs6432223	TRANSFAC, JASPAR	1.13E-04

RegSNVs that were predicted to increase ER binding in MCF7 cells and had an E2-regulated gene within 5 kb were selected. This table shows the top ten candidates showing the most significant differential binding between WT and variant alleles

### An intronic regSNV in *IGF1R* controls ER binding and activity in an allele-specific manner

**Fig. 2:**
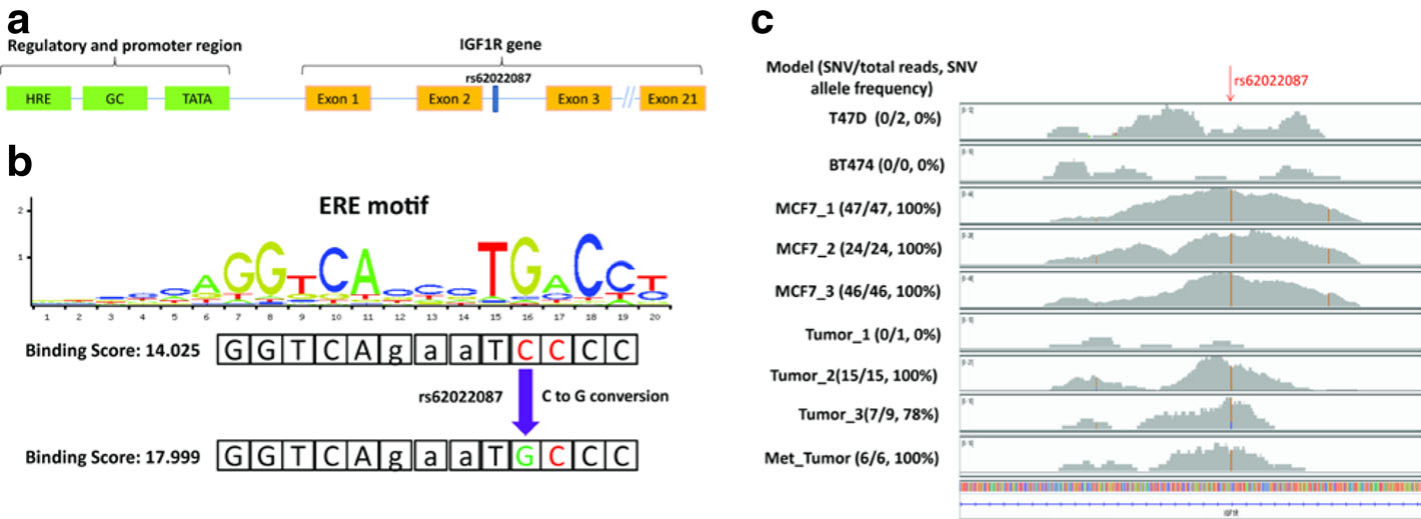
The location of rs62022087 in genome and ER ChIP-binding sites. **a** *Schematic view* of DSV genomic position in IGF1R gene. **b** The position of IGF1R SNP with regards to canonical ERE sequence. Binding score was calculated by Jaspar database tool (http://jaspar.genereg.net). **c** The distribution of ER ChIP-seq reads flanking rs62022087 SNP in different cell line models as well as patient tumors [1]. The numbers in *parentheses* are the fraction and percentage of the reads containing mutant allele, respectively

Our motif assessment analysis showed that rs62022087 is one of the top three regSNVs putatively modulating ER binding to an ER-regulated gene. This SNV is located within an ERE and the G of the SNV was predicted to alter the ERE from a weak to a strong binding site (Fig. 2a) (*p* value = 2.03E-05). rs62022087, with a minor allele frequency (MAF) of 13.5%, is located centrally in the second intron of *IGF1R* (Fig. 2b), which is a region hosting several active histone marks such as H3K29ac and H3k4Me1, and a number of transcription factors including FOXA1, FOXA2, and E2F1, and finally DNase I hypersensitive sites (Additional file 2: Figure S1). Direct genotyping of rs62022087 by Sanger sequencing of MCF7 genomic DNA indicated that the locus is heterozygous in contrast to T47D, ZR75, and BT474 cells. We examined whether ChIP-seq data showed an allelic preference towards the regSNV, as would be predicted from the increased ERE motif binding [1]. Supporting this, cell lines (MCF7) and human breast tumors (Tumor_2, Tumor_3, and Met_Tumor, extracted from [1]) which harbor the regSNV showed increased ER ChIP-seq reads in this ER ChIP-binding site (Fig. 2c). In addition, the allele frequency of rs62022087 is strongly biased towards the variant allele in the samples carrying the regSNV (MCF7: 100%, Tumor 2: 100%, Tumor 3: 78%, Met Tumor: 100%, derived from [1]), further supporting the concept that the regSNV results in increased ER binding. A similar phenomenon was observed in the ChIP-seq datasets of two other studies (Additional file 2: Figure S2). rs62022087 genotype in T47D, BT474, and Tumor 1 is WT whereas it is heterozygous in MCF7, Tumor 2, and Tumor 3. Collectively, these data suggest that ER has higher affinity for the regSNV allele compared to the wild-type allele.

**Fig. 3:**
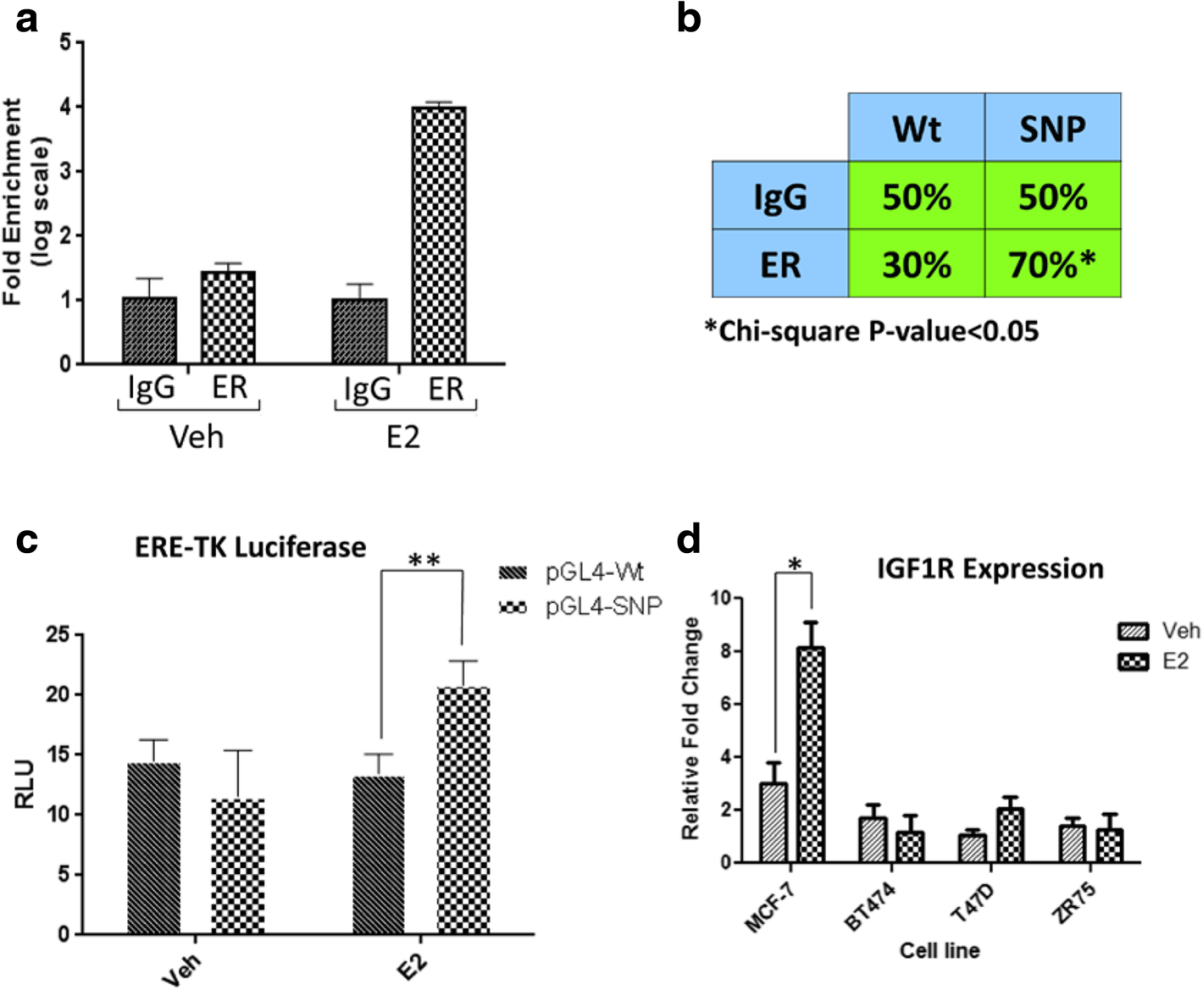
IGF1R SNP can affect ER binding and result in higher gene expression. **a** Confirmation of ER binding to IGF1R SNP by ChIP-qPCR in MCF7 cell line. The cells were estrogen deprived for 3 days and subsequently treated by Veh or E2 (1nM) for 45 min. ChIP was performed as describes in the “Methods” section. ER binding is significantly enriched upon treatment by E2. **b** Allele-specific ChIP result shows a significant enrichment of SNP allele (70%) vs. WT allele (30%) in ER ChIP-binding site. **c** Luciferase transactivation assay using MCF7 cells transfected with constructs containing the ER ChIP-binding site with WT or SNP. The luciferase assay demonstrates that the binding site with variant allele has higher affinity to ER upon induction by estradiol (1 nM) (**p value < 0.01). **d** IGF1R gene expression in different breast cancer cell lines treated by Veh or E2 (1 nM). The significant induction of IGF1R expression in MCF7 cell line may contribute to the presence of regulatory SNP compared to the other cell lines with WT allele (*p value < 0.05)

We next performed experiments to directly examine the role of the regSNV in altering ER-mediated induction of IGF1R expression. ER ChIP-qPCR in MCF7 cells showed that ER bound the genomic region containing regSNV in intron 2 of *IGF1R* with a fourfold enrichment following E2 treatment (Fig. 3a). Allele-specific ChIP showed a significant enrichment of the regSNV allele (G allele) in the DNA bound to ER (Fig. 3b). Cloning of the ER ChIP-binding site (with or without the regSNV site) upstream of a heterologous promoter and luciferase indicated that the ER ChIP-binding site containing the regSNV showed greater ER-induced luciferase expression upon estradiol treatment (Fig. 3c). This indicates that the G allele is more potent in recruiting ER and subsequently leading to increased induction of IGF1R expression (Fig. 3d). Consistent with this, estradiol induced IGF1R expression greater in MCF7 cells compared to the cell lines that lack the regSNV and are homozygous for the wild-type allele. Taken together, our in vitro experiments validate that one of the top computational regSNV predictions (rs62022087) favors ER binding and results in elevated estradiol induction of IGF1R expression.

### Discovery of regSNVs in ER ChIP-seq data from breast cancer

We next applied our workflow to all available ER ChIP-seq data in breast cancer cell lines and tumors comprising a total of 43 datasets from seven independent studies (Additional file 1: Table S5–13) [1, 20, 22–25, 29–31] (GEO numbers provided in “Methods”). RegSNVs were identified within ER ChIP-binding sites and the closest genes to the regSNVs annotated. The genomic position of the regSNVs was annotated based on where they are located in the genome (e.g., exonic, intronic, etc.). Additional file 2: Figure S3 shows the distribution of regSNVs in the analyzed models from available ER ChIP-seq data. The majority of regulatory variants are located in intergenic areas whose functionality is not well-characterized. Many SNVs are also in intronic areas, suggesting a major role of introns in estrogen regulation of the gene expression. This is not surprising as the majority of ER ChIP-binding sites lie in intergenic and intronic segments of the genome.

To examine the function of these regSNVs, we determined whether their presence was associated with altered gene expression using data from 1045 samples in TCGA. RegSNVs (*n* = 11,605) are enriched in the proximity of genes differentially regulated between ER+ (*n* = 808) and ER– tumors (*n* = 237) (Chi-square test, *p* value < 0.01), suggesting a role of these SNVs in estrogen response. Further, to determine if the regSNVs have a functional role, we assessed the correlation of genotype (i.e., regSNV) with neighboring gene expression. Out of 11,605 regSNVs with dbSNP rsIDs, we found 9082 to be present in TCGA SNP array data. We used these data to find the samples with the SNVs and then compared the expression of target genes in SNV versus WT carriers in only ER+ samples. This led to the discovery of 17 regSNVs associated with the expression of their adjacent genes (adjusted *p* value < 0.01, Table 2). Of these, there was sufficient coverage in the ChIP-seq data (>10 reads) to call allele-specific binding for six. All six showed greater than 50% of reads containing the allele with the regSNV, suggesting that the SNVs increase ER binding, as predicted by our pipeline (Additional file 1: Table S14). The majority of the regSNVs (13 out of 17) were located in the promoter of target genes, further showing that they are likely to be functional (Table 2). All the 17 regSNVs were queried at the GWAS catalog database (http://www.ebi.ac.uk/gwas) but no association was found with breast cancer or ER biology in GWAS.

**Table 2:** List of regSNVs associated with the expression of their target genes in TCGA primary tumors

RegSNV ID	Location	Target gene	No. of tumors with SNV genotype (*n* = 501)	log2 fold change	Adjusted *p* value
rs36208869	Promoter	GSTM1	32	4.58	1.25E-08
rs1131017	Promoter	RPS26	318	–0.39	5.19E-07
rs7113753	Promoter	TRAPPC4	180	0.26	2.79E-05
rs1412825	Promoter	LRRIQ3	243	–0.22	3.64E-05
rs34282253	Promoter	XKR9	119	0.41	4.62E-05
rs10747783	Promoter	TSFM	205	–0.22	0.000157917
rs252923	Promoter	SETD9	197	0.41	0.000157917
rs41293275	Promoter	NSUN4	175	–0.22	0.000241865
rs3213745	Promoter	CEBPZ	241	–0.19	0.000444457
rs2732649	intergenic	LRRC37A	132	0.12	0.002214471
rs17361749	Promoter	NSUN4	168	–0.2	0.002736821
rs10489769	Promoter	NSUN4	172	–0.19	0.004515197
rs10956142	intergenic	ANXA13	38	–0.29	0.004515197
rs2939587	Promoter	TM2D3	260	0.21	0.005471564
rs1291363	Promoter	HTR7P1	315	0.59	0.006560413
rs4418583	Intron	LDLRAP1	248	0.24	0.006560413
rs3811254	Intron	OR4E2	3	0.04	0.009385423

The tumors containing regSNVs were identified using SNP genotyping and the expression of target genes were compared between WT and variant carriers. This table shows the top regSNVs significantly regulating their corresponding target genes (adjusted *p* value < 0.01)

**Fig. 4:**
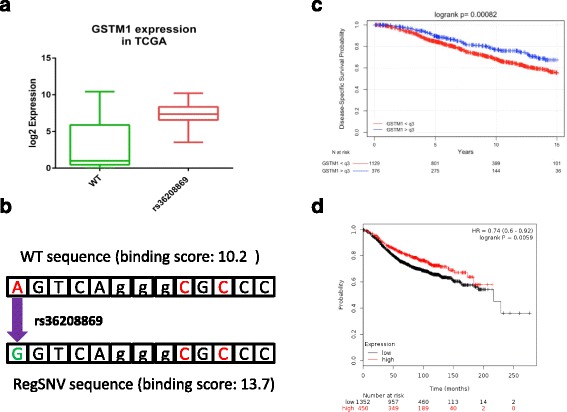
The expression of GSTM1 in ER+ TCGA breast tumors (*n* = 385) is higher in rs36208869 carriers (*n* = 32) compared to WT carriers (*n* = 353). **a** The SNP array and RNA-seq data from TCGA were used for this comparison. Tumors containing the regSNV show significantly higher expression of GSTM1 compared to those with WT allele (adjusted *p* value = 1.25E-08). **b** The position of rs36208869 relative to the ERE located in the promoter of GSTM1. Binding score was calculated by Jaspar database tool (http://jaspar.genereg.net) **c** Disease-specific survival of ER+ patients from METABRIC (*n* = 1505) separated by upper quartile (q3) expression of GSTM1 (Illumina HT-12 v3 platform, probe: 1762255). **d** Recurrence-free survival of ER+ patients from the KM-plotter dataset (*n* = 1802) separated by upper quartile expression of GSTM1 (Affymetrix platform, probe: 204550_x_at)

The top candidate in our list is rs36208869 which is an SNV in the promoter of Glutathione S-Transferase Mu 1 gene, *GSTM1*. Our algorithm predicted an increased binding of ER to the SNP allele and we observed an approximately 16-fold higher expression in tumors carrying the SNP (adjusted *p* value = 1.25E-08) (Fig. 4a, b). GSTM1 encodes for a member of the glutathione S-transferase family which is responsible for detoxification of chemical compounds including carcinogens and products of oxidative stress [32]. A large body of evidence has shown that loss of GSTM1 increases the susceptibility to several types of cancer including lung and bladder [33–35]. Interestingly, we examined the METABRIC and KM-Plotter datasets and found that higher expression of GSTM1 in breast tumors is associated with better survival of patients with ER+ tumors (Fig. 4c, d, logrank *p* value for METABRIC = 8.2E-4, logrank *p* value for KM-Plotter = 5.9E-3).

## Discussion

Global genetic variation in TF-binding sites can lead to widespread changes in gene expression among different individuals [36–38]. Analyzing complete genomes of different cancer types has elucidated recurrent mutations in the genomic regions potentially regulated by TFs [39–41]. However, deciphering how genome-wide DNA variants affect TF binding remains understudied. We present a computational framework, which analyzes ChIP-seq reads to identify regSNVs in TF-binding sites. We used this strategy, in combination with experimental studies, to validate the impact of regSNVs on corresponding DNA motifs. While other studies have identified regSNVs in ER ChIP-binding sites using a biased approach involving genotyping information from resources such as dbSNP and GWAS [5, 9, 10], our approach differs by identifying SNVs directly from ChIP-seq data, thus increasing the likelihood of identifying novel regSNVs in TF-binding sites.

The MCF7 cell line is one of the most studied models for understanding ER biology and results from this cell line have had a fundamental impact upon breast cancer research and patient outcome [42]. Using available ER ChIP-seq data in MCF7, we investigated the genetic variation in ER ChIP-binding sites with this model. The number of binding sites varies significantly between the MCF7 datasets in the range of 15,677–79,978 sites. This high degree of variation may be due to slight differences in technical details, such as culturing conditions or cell line passage numbers, utilized for the ChIP experiments. We used an overlap of ER ChIP-binding sites for this study. Our analysis revealed a functional regSNV (rs62022087) in intron 2 of the *IGF1R* gene which was predicted to increase ER binding. We show that the rs62022087 SNP results in increased ER recruitment to intron 2 and increased E2-mediated expression of IGF1R gene in MCF7 cells compared to cell lines carrying the WT allele. IGF1R overexpression has been implicated to play an important role in the development of breast cancer [43–45] and the crosstalk between IGF1R and estrogen signaling has been well established in malignant breast tissue [46–48]. Furthermore, several coding and non-coding polymorphisms have been shown increase the susceptibility to breast cancer [49, 50]. This prompted us to obtain more information on this SNP from GWAS and correlate it with clinical outcome in breast cancer patients. However, neither rs62022087 nor any of the SNPs in LD with our candidate SNP are genotyped by Affymetrix chips, which are commonly used in GWAS and TCGA data. Further sequencing studies in large cohorts are warranted to characterize the potential role of this regulatory SNP in development and progression of breast cancer.

Our computational framework is able to detect not only germline variants, but also rare somatic mutations which may alter the affinity of TF to DNA. However, the general low coverage of ChIP-seq data makes it challenging to perform accurate variant calling. Therefore, in this study we pooled the reads from multiple datasets on the same cell line to improve the confidence of calls. With the decreased costs of sequencing, we expect that increased coverage in ChIP-seq studies will alleviate this problem in the near future.

Applying our pipeline to all available ER ChIP-seq data characterized thousands of regSNV candidates in multiple breast cancer models, which may potentially change the binding of ER. About 96% of these variants are annotated in the dbSNP and 1000 Genome databases and are thus likely to be germline alterations, however, we did not have access to normal matched samples to confirm this. This high rate of germline SNPs may reflect our inability to detect low allele frequency somatic mutations due to the low read coverage of ChIP-seq data. The majority of regSNVs reside in intronic regions of the genome, similar to the regSNV we have characterized in intron 2 of the *IGFIR* gene. Several studies have identified regulatory SNPs in genes associated with breast cancer susceptibility and treatment [4–6, 51]. By integrating multi-omics large datasets, we found 17 regSNVs associated with the expression of adjacent genes. The top candidate was a SNP in the promoter of GSTM1 whose expression is associated with survival in breast cancer patients. ChIP-seq reads provided further evidence showing the variant allele is enriched in the ER ChIP-binding sites although we were not able to infer the true reference genotype due to not having access to normal tissue information in analyzed samples (Additional file 1: Table S14). Several studies have shown coding and non-coding polymorphisms in GSTM1 could modify the risk for breast cancer suggesting the importance of this gene in this disease [52–55].

The role of non-coding genomic variants in cancer and other diseases has been largely understudied due to the technological challenges and lack of understanding about the non-coding genome. In this paper, we present a novel pipeline to identify regulatory SNVs by integrating multi-omics data and validate them through in vitro studies. Our methodology is applicable to not only other types of cancer, but also other genetic based diseases. The screen for impactful regulatory variants will soon become part of genetic testing as our knowledge of non-coding genome improves and sequencing costs are reduced. Such genetic tests are of great importance to public health in order to tailor the treatment to the needs of each individual patient.

## Conclusions

In this study, we developed a pipeline to identify potential regSNVs in ER ChIP-binding sites which may have downstream transcriptomic changes and therefore, confer phenotpyic impact in ER+ breast cancer. By integrating ChIP-seq, gene expression, and patient survival data in breast cancer, we were able to link regSNVs that may potentially cis-regulate target genes and may have prognostic value. We found an intronic SNV in IGF1R is capable of promoting ER binding on DNA and increases the expression of IGF1R gene. Simlarly, a regSNV in the promoter of GSTM1 gene, rs36208869, was predicted in our pipeline to increase ER binding and was shown to be highly correlated with the expression of GSTM1 whose higher levels in ER+ breast tumors are associated with a better survival. Our findings highlight the role of non-coding regulatory variants in modulating ER binding that may have prognostic value and need to be further studied in the clinical settings.

## Additional files

Additional file 1: Table S1. The list of all ER ChIP-seq datasets in breast cancer. **Table S2.** List of E2-regulated genes common in vitro, in vivo, and TCGA. **Table S3.** List of the studies used for the identification of E2-regulated genes. **Table S4.** Primer sets used for different assays. **Table S5.** The list of regulatory SNVs in MCF7 Cell line. **Table S6.** The list of regulatory SNVs in BT474 cell line. **Table S7.** The list of regulatory SNVs in MDA-MB-134 cell line. **Table S8.** The list of regulatory SNVs in T47D cell line. **Table S9.** The list of regulatory SNVs in TAMR cell line. **Table S10.** The list of regulatory SNVs in ZR75 cell line. **Table S11.** The list of regulatory SNVs in good prognosis tumors. **Table S12.** The list of regulatory SNVs in bad prognosis tumors. **Table S13.** The list of regulatory SNVs in metastatic tumors. **Table S14.** The allele frequency of top RegSNVs in ER-binding sites with sufficient coverage. (XLSX 3641 kb)
Additional file 2: Figure S1. The UCSC gnome browser view of the second intron in IGF1R gene. The index SNP, rs62022087, seems to be located in a region bound by several chromatin-modifying factors based on ENCODE data. **Figure S2.** The visualization of ChIP-seq reads from multiple cell lines over rs62022087 SNP site in two individual studies: (**A**) Hurtado et al. {Hurtado, 2011 #23}, (**B**) Joseph et al. {Joseph, 2010 #15}. **Figure S3.** The distribution of RegSNVs over the gnome across a panel of breast cancer cell lines, good and bad prognosis tumors. The binding sites from different ER ChIP-seq datasets were extracted and annotated based on their location in the genome. The majority of the binding sites are located in the intergenic and intronic areas. (DOCX 384 kb)

## Abbreviations

ChIP-seq: Chromatin immunoprecipitation sequencing
ER: Estrogen receptor
ERE: Estrogen response elements
MAF: Minor allele frequency
METABRIC: Molecular Taxonomy of Breast Cancer International Consortium
PGRR: Pittsburgh Genome Resource Repository
SNP: Single nucleotide polymorphisms
SNV: Single nucleotide variants
TCGA: The Cancer Genome Atlas
TF: Transcription factor
TPM: Transcripts per million
WT: Wild-type
