# Supplementary material for: Non-coding single nucleotide variants affecting estrogen receptor binding and activity
Source: Genome Med. 2016 Dec 13;8:128. doi: 10.1186/s13073-016-0382-0 (PMC5154163; doi:10.1186/s13073-016-0382-0)
Supplement: Additional file 2: Figure S1. — The UCSC gnome browser view of the second intron in IGF1R gene. The index SNP, rs62022087, seems to be located in a region bound by several chromatin-modifying factors based on ENCODE data. Figure S2. The visualization of ChIP-seq reads from multiple cell lines over rs62022087 SNP site in two individual studies: (A) Hurtado et al. {Hurtado, 2011 #23}, (B) Joseph et al. {Joseph, 2010 #15}. Figure S3. The distribution of RegSNVs over the gnome across a panel of breast cancer cell lines, good and bad prognosis tumors. The binding sites from different ER ChIP-seq datasets were extracted and annotated based on their location in the genome. The majority of the binding sites are located in the intergenic and intronic areas. (DOCX 384 kb) [file 13073_2016_382_MOESM2_ESM.docx]

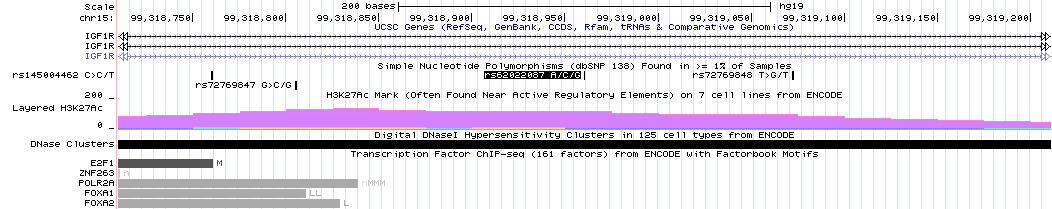
**Figure S1.** **The UCSC gnome browser view of the second intron in IGF1R gene.** The index SNP, rs62022087, seems to be located in a region bound by several chromatin modifying factors based on ENCODE data.


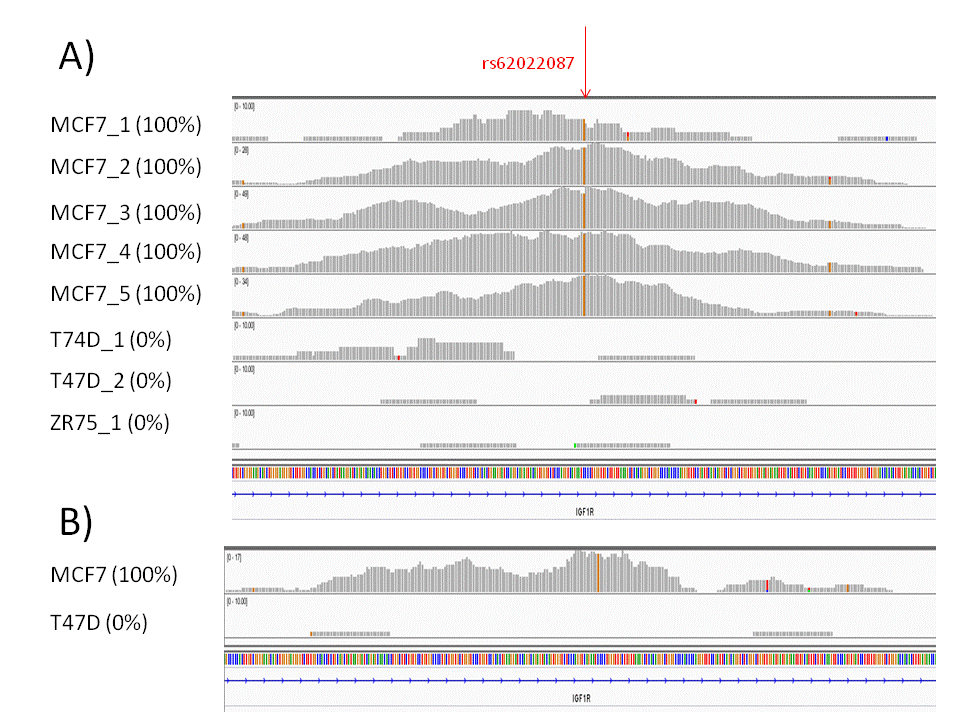


**Figure S2.** The visualization of ChIP-seq reads from multiple cell lines over rs62022087 SNP site in two individual studies: A) Hurtado A. *et al*. {Hurtado, 2011 #23} B) Joseph R. *et al*. {Joseph, 2010 #15}


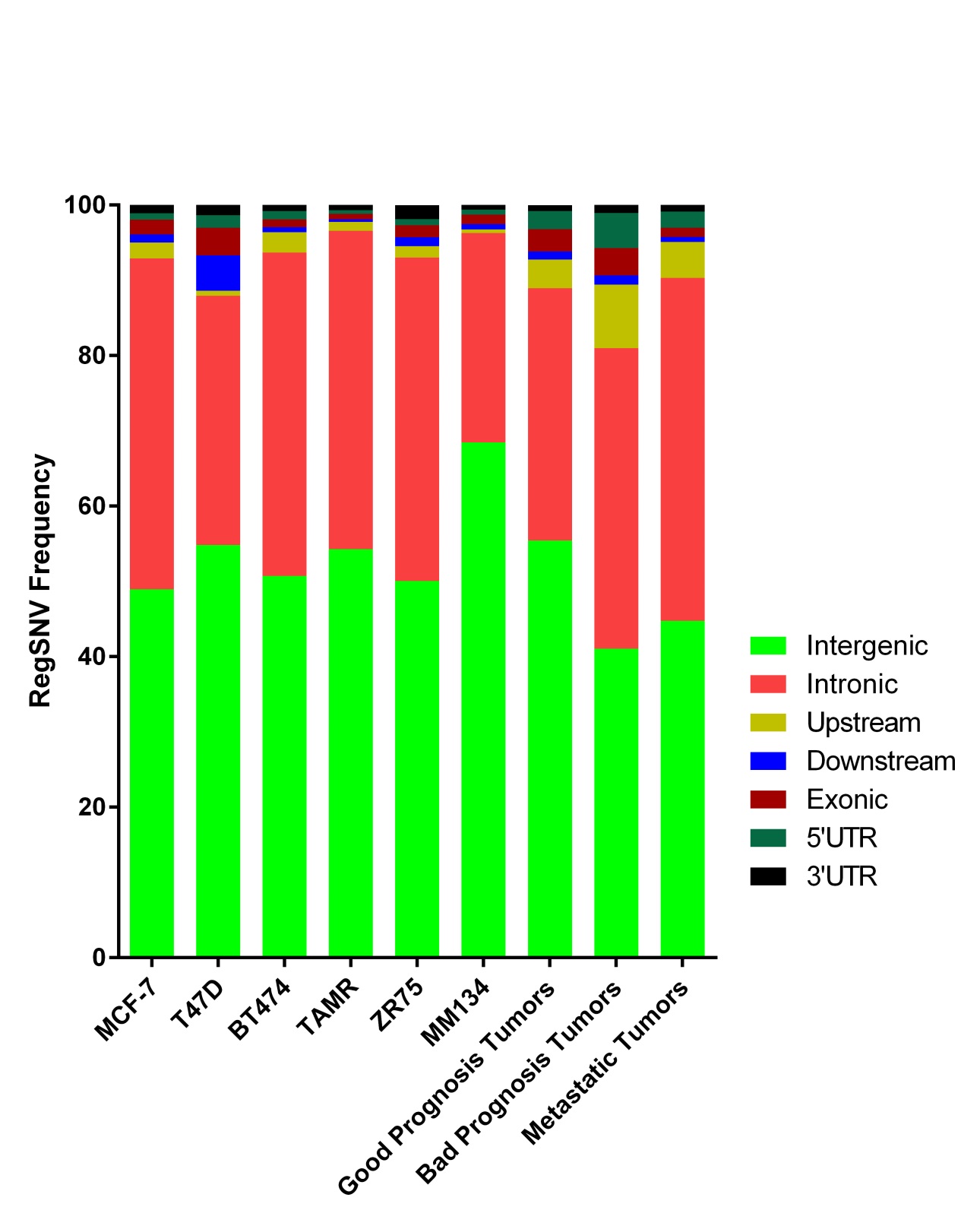


**Figure S3.** **The distribution of RegSNVs over the gnome across a panel of breast cancer cell lines, good and bad prognosis tumors.** The binding sites from different ER ChIP-seq data sets were extracted and annotated based on their location in the genome. The majority of the binding sites are located in the intergenic and intronic areas.
